# Microbiome diversity, composition and assembly in a California citrus orchard

**DOI:** 10.3389/fmicb.2023.1100590

**Published:** 2023-02-22

**Authors:** MengYuan Xi, Elizabeth Deyett, Jason E. Stajich, Ashraf El-Kereamy, M. Caroline Roper, Philippe E. Rolshausen

**Affiliations:** ^1^Department of Botany and Plant Sciences, University of California, Riverside, Riverside, CA, United States; ^2^Department of Microbiology and Plant Pathology, University of California, Riverside, Riverside, CA, United States

**Keywords:** Huanglongbing, biofertilizers, rhizosphere, citrus flush, caulosphere, anthosphere, biopesticides, endophytes

## Abstract

The citrus root and rhizosphere microbiomes have been relatively well described in the literature, especially in the context of Huanglonbing disease. Yet questions addressing the assembly of root microbial endophytes have remained unanswered. In the above ground tree tissues, leaves and stems have been the research focus point, while flush and flower microbiomes, two important tissues in the vegetative and reproductive cycles of the tree, are not well described. In this study, the fungal and bacterial taxa in five biocompartments (bulk soil, rhizosphere, root endosphere, flower and flush) of citrus trees grown in a single California orchard were profiled using an amplicon-based metagenomic Illumina sequencing approach. Trees with no observable signs of abiotic or biotic stresses were sampled for two consecutive years during the floral development phase. The rhizosphere was the most biodiverse compartment compared to bulk soil, root endosphere, flower and flush microbiomes. In addition, the belowground bacteriome was more diverse than the mycobiome. Microbial richness decreased significantly from the root exosphere to the endosphere and was overall low in the above ground tissues. Root endophytic microbial community composition shared strong similarities to the rhizosphere but also contained few taxa from above ground tissues. Our data indicated compartmentalization of the microbiome with distinct profiles between above and below ground microbial communities. However, several taxa were present across all compartments suggesting the existence of a core citrus microbiota. These findings highlight key microbial taxa that could be engineered as biopesticides and biofertilizers for citriculture.

## Introduction

Developing integrated agriculture systems has become increasingly needed in the face of mounting global challenges. The environmental impact of agrochemical pesticides and fertilizers is leading to changes in consumer behavior toward sustainably grown food and food products and as a result, farmers are increasingly relying on biological-based technologies and less on synthetic chemistries. Microbiomes have been shown to provide many benefits to plants by priming the immune system and protecting them from diseases, facilitating nutrient acquisition, and overall enhancing health and increasing yield. Taking advantage of the microbiome at work, i.e., the capitalization on microbial traits that are beneficial to the host or the environment or both, presents a promising avenue for the development of a more sustainable next-generation agriculture (Schlaeppi and Bulgarelli, 2015).

Commonly occurring organisms across similar microbiomes form a core microbial community that is hypothesized to play critical roles in ecosystem functioning within that type of microbial habitat (Shade and Handelsman, 2012; Gopal et al., 2013). While many deep sequencing studies have shown that plant microbiomes are made up of a plethora of microbial taxa, only a few taxa typically predominate in the larger community (Sagaram et al., 2009; Gottel et al., 2011; Weinert et al., 2011; Bodenhausen et al., 2013; Peiffer et al., 2013). Even in a variety of experimental settings, some of the highly abundant taxa in these studies are noticeably conserved across the microbiomes of related plant species. This implies that a core microbial community consistently associates with specific hosts at different spatial and temporal scales. However, it is known that the composition of the plant microbiota is influenced by a number of biotic and abiotic factors (Redford and Fierer, 2009; Bulgarelli et al., 2012; Lundberg et al., 2012; Rastogi et al., 2012). There is still much to learn about the composition of the core microbiome community and its significance for plant health, given that only a few studies have identified the key players in plant-associated microbial communities (Bulgarelli et al., 2012; Lundberg et al., 2012; Rastogi et al., 2012).

Citrus is one of the most important perennial fruit crops in the world. Being a good source of vitamins, fiber, and minerals, it is commended for its nutritional qualities and advantages for human health. Citrus is also a major contributor to the economic value of the agricultural sector. It accounts for 16% of the total value of the United States fruit production (Li et al., 2020) with California representing 80% of the nation’s fresh fruit market with an annual value of 2.3 billion dollars (CDFA, 2021). There has been tremendous interest in exploring the structure and function of the citrus phyllosphere and rhizosphere microbiomes and engineering its assembly to address current challenges in citriculture (Zhang et al., 2021; Ginnan et al., 2022). Root microbiome has emerged as a focal point of citrus health especially in the context of Huanglongbing (HLB) disease (Blaustein et al., 2017; Xu et al., 2018; Ginnan et al., 2020; Wu et al., 2020).

Huanglongbing (HLB) or citrus greening is considered the most serious problem of citrus worldwide (National Research Council, 2010). HLB is caused by an uncultivable Gram-negative phloem-limited bacteria belonging to the *Candidatus* Liberibacter species (i.e., *Ca.* L. asiaticus, CLas; *Ca.* L. africanus and *Ca.* L. americanus), which are transmitted from infected to healthy plants by citrus psyllids (Bové, 2006). *C*Las infection causes phloem sieve occlusion and impairs translocation of photo-assimilated carbon to the root zone thereby weakening trees by decreasing the energy pool of non-structural carbohydrates (Etxeberria et al., 2009). Lasting infection leads to root collapse and dysbiosis of root associated microbial communities including depletion of keystone taxa and enrichment of saprobes and parasitic soilborne fungi such as *Fusarium* and *Phytophthora* (Ginnan et al., 2020). However, despite our better understanding of the importance of root health on disease management, tree health and productivity, gaps remain to confidently develop effective guidelines for long-term disease management.

The highest concentrations of *C*Las can be found in midribs of flush (Chiyaka et al., 2012). A flush shoot may be defined as a new shoot growth with immature leaves but can range from as small as newly breaking buds of just feather flush to fully elongated shoots with expanded, tender leaves. In California and Mediterranean climates, flush is produced twice annually in relatively well-defined cycles, one related to plant growth in summer-autumn, and one related to flowering and fruiting in spring. Timing of flush development is genetically and environmentally governed, with temperature, photoperiod, solar radiation and rainfall (Moss, 1969, 1976; Olesen et al., 2013). The most critical of these for fruit production is the spring leaf flush since it coincides with both flowering and early fruit development. However, the microbial composition of the citrus flush has to our knowledge not been elucidated, even though this tissue is at the forefront of the infection in the HLB pathosystem. Profiling the citrus flush microbiome could identify potential beneficial organisms that are inhibitory to *C*Las or provide the host with environmental fitness and horticultural advantage.

Similar to the flush, the study of flower microbiome has surprisingly received little attention despite its direct role in fruit production. In citrus, flowering time and abundance depend largely on the species, the tree age, and the climatic conditions (Lau and Lennon, 2011; Agustí et al., 2020). However, research indicated that rhizosphere microbiome can also drive changes in the host phenological traits including flowering period (Lau and Lennon, 2011; Lu et al., 2018). The host phenological stage appears to be a major driver of the leaf microbiome assemblage indicating that it could also influence flower microbiome composition (Ginnan et al., 2022). Flowering is the most important determinate of yield and quality of citrus fruit production (Stander, 2015). Particularly, flowers provide ephemeral but unique nutrient-rich and protective habitats for microorganisms (Aleklett et al., 2014) and the microbial make-up of flowers may affect disease outcome and in turn fruit yield. For example, fire blight disease severity of apple blossoms caused by *Erwinia amylovora* can be mitigated by treating flowers with endogenous microbial taxa (Cui et al., 2021). The understanding of the reproductive microbiome function on flowering may hold the key to enhance productivity in agroecosystems.

The objective of this study was to fill in the knowledge gap about root microbial assemblage in citrus to better identify key microbes recruited by the host that likely harbor beneficial properties, increase the host environmental fitness, and support tree health. In addition, our goal was to profile the microbiome of the flower and the flush, two young tissues that had not been extensively studied despite their critical importance in the tree vegetative and reproductive cycles. Flush is also critical to the HLB disease epidemiology. Here, we provide a microbial map of five distinct compartments (bulk soil, rhizosphere, root endosphere, flush and flower) of citrus from a single orchard over a two-year period and discuss in what capacity the information acquired with this research may help citrus production.

## Materials and methods

### Plant sampling and processing

The experimental orchard is located at the Lindcove Research and Extension Center, California (GPS coordinates 36°21′10″N; 119°03′40″W). The plant materials were collected from 11 years-old conventionally farmed citrus cv. ‘Tango’ on ‘Carrizo’ rootstock (*Citrus sinensis* L. Osbeck × *Poncirus trifoliata* L.). All samples were collected at the flower initiation stage on 4/7/2021 and 3/28/2022. Flower, flush (the new foliar growth between bud break and shoot expansion), root and rhizosphere samples were collected each year from 12 random trees. Flush and flower samples were collected from the four quadrants and pooled. Feeder roots were sampled from two sides of the tree approximately 0.3 m away from the base of the trunk. Five bulk soil samples were also collected each year from the four corners and the middle of the citrus grove. Gloves were changed and clippers and shovels were sterilized with 30% household bleach between each sampled tree. All samples were immediately placed on ice in a cooler for transit to the laboratory and were frozen. All samples were processed within 24 h. Root and rhizosphere samples were processed as described by (Lundberg et al., 2012). Briefly, roots were placed in sterile 50-mL conical tube with 25-mL of PBS with 200-μL L^−1^ Silwet^®^ L-77 surfactant. Samples were vortexed at maxim speed for 15 s. Roots were then transferred to a clean 50-mL conical tube with 25 ml of PBS. The first tube was centrifuged at 3200 *g* for 15 min and the aqueous layer was removed. The pellet was retained as the rhizosphere fraction. The roots continued to be vortexed and were moved to a clean PBS tube until PBS remained clear after vortexing. Roots were then sonicated using a Branson Sonifier 450 at a low frequency for 5 min (five 30 s bursts followed by 30 s breaks). Roots were then stored at −70°C for further processing. Flowers, flushes, and roots were then lyophilized in the FreeZone 2.5-L benchtop freeze dry system (Labconco, Kansas City, United States) for 72 h. Specifically, flower and flush samples were not surface sterilized; thus, the aboveground microbial next-generation sequencing datasets included both epiphytes and endophytes. Samples were then ground to a powder using the MM300 grinder (Retsch, Haan, Germany) in a 35-mL stainless steel grinding jar with 20-mm stainless steel balls at 25 oscillations per second in 30-s increments until sample was fully pulverized.

### Microbiome library preparation

DNA was extracted from all samples using the ZymoBIOMICS DNA miniprep kit per manufacturer’s protocol, using 100 mg of dried tissue or 250 mg of wet rhizosphere (Zymo Research, Irvine, United States). DNA was assessed for quality and quantity using the Qubit 4 Fluorometer with the Qubit dsDNA HS Assay (Thermo Fisher Scientific Inc., MA, United States). Both bacterial 16S–V4 and fungal ITS rRNA regions were amplified using the Earth Microbiome protocol and primers.^1^ Briefly, primers 515F(GTGYCAGCMGCCGCGGTAA) and 806R (GGACTACNVGGGTWTCTAAT) were used for bacterial microbiomes and ITS1f (CTTGGTCATTTAGAGGAAGTAA) and ITS2 (GCTGCGTTCTTCATCGATGC) for fungal ITS amplification (Caporaso et al., 2010). PCR reactions of 25 μl contained 10 μl of Phusion hot start flex 2 × master mix, 0.5 μl of each primer (10 μm) and 2 μl of DNA. In bacterial above-ground tissue (flower and flush), universal pPNA and mPNA clamps were added at a starting concentration of 1.25 μl (5 μm). These clamps were designed to reduce the amplification of host chloroplasts and mitochondria while having no effect on bacterial amplification (Fitzpatrick et al., 2018b). A negative control was added to each PCR to ensure barcodes and master mix were not contaminated. Successful amplification was verified on a 1% agarose gel and DNA was quantified using the NanoDrop 2000 Spectrophotometers (Thermo Fisher Scientific Inc., MA, United States). A total of 1,200 ng of each sample in a library were combined into an Eppendorf tube and cleaned using the AMPure XP PCR purification system (Beckman Coulter, Brea, United States) per manufacturer’s protocol. Final concentration of libraries was determined using both qPCR and bioanalyzer before being sequenced on the MiSeq instrument (Illumina, San Diego, United States) using Miseq run (2 × 300 paired end) for fungal reads and Miseq run (2 × 250 paired end) for bacterial microbiome at the UC Riverside Genomics Core facility. Fungal and bacterial sequences were deposited in NCBI under the accession number SUB12502574 and SUB12495203, respectively.

### Computational analysis

The R Core Team v4.1.1 was used to perform all computational analysis. Most processing for the reads were done in DADA2 v 1.16.0 (Callahan et al., 2016) including further quality control sequencing filtering, dereplication, chimera identification, merging paired end reads, and construction of sequence tables. Taxonomy identification was assigned using the SILVA SSU r138.1 reference database for bacterial taxa and Unite database v 10.5.2021 for fungal taxa. Phyloseq v 1.36.0 (McMurdie and Holmes, 2013) and ggplot2 v3.3.5 packages (Wickham, 2016) were used for much of the graphical and statistical analyzes of the data. Unidentified microbes at the kingdom or phylum level, or microbes that occurred less than two times within all 24 trees (12 tree samples per year) were removed from the full dataset. The bacterial dataset totaled 106 samples (24 flower, 24 flush, 24 rhizosphere, 24 root and 5 soil samples) and the fungal dataset totaled 104 samples (23 flower, 24 flush, 24 rhizosphere, 23 root and 5 soil samples) after filtering out poor quality reads, chloroplast, mitochondria, taxa with unidentified phyla. After removal of singletons and doubletons, the total ASVs were of 10,483 (soil = 4,395; rhizosphere = 7,635; root = 1997; flush = 129; flower = 128) and 5,155 (soil = 707; rhizosphere = 2,964; root = 1,333; flush = 860; flower = 905) for the bacterial and fungal datasets, respectively. Shannon diversity index was used as a metric of taxa diversity within the communities. Kruskall–Wallis and pairwise Wilcoxon rank sum tests were run to verify statistical differences among groups. Phylum bar charts and genus bar charts were constructed by aggregating taxa at the phylum level and genus level, respectively. Samples were also constructed by tissue compartments and transforming to relative abundance. Bray–Curtis dissimilarity was used to calculate the compositional similarities between samples and was visualized with NMDS (Non-metric MultiDimenstional Scaling) plots using the Vegan package v 2.5-7. To determine statistical significance of beta diversity, Adonis tests were run. Venn diagrams were created using UpSetR v 1.4.0 by transforming to relative abundance and filtering taxa to those that occur greater than 0.1% and are prevalent in at least two samples of that tissue type. Data was aggregated by genus and transformed to relative abundance for the prevalent Venn diagrams. Taxa were denoted as prevalent in each biocompartment. Graphs were generated using VennDiagram v1.6.20. Data was aggregated to the ASV or genus level and transformed to relative abundance for the concentric pie charts representing core microbiome. ASVs/genera were filtered based on core microbiome as previously defined. DeSeq2 v 1.30.1 was utilized and visualized using Pheatmap v1.0.12 to find microbes associated with a biocompartment and above-and belowground sections. Genera were filtered by relative abundance, *p* value and log2 fold change, keeping only genera occurring at ≥ 1% relative abundance of whole dataset with *p* < 0.01 and having a log2 fold change > 5 or < −5. Heat maps represent the relative abundance of the data.

## Results

**Figure 1:**
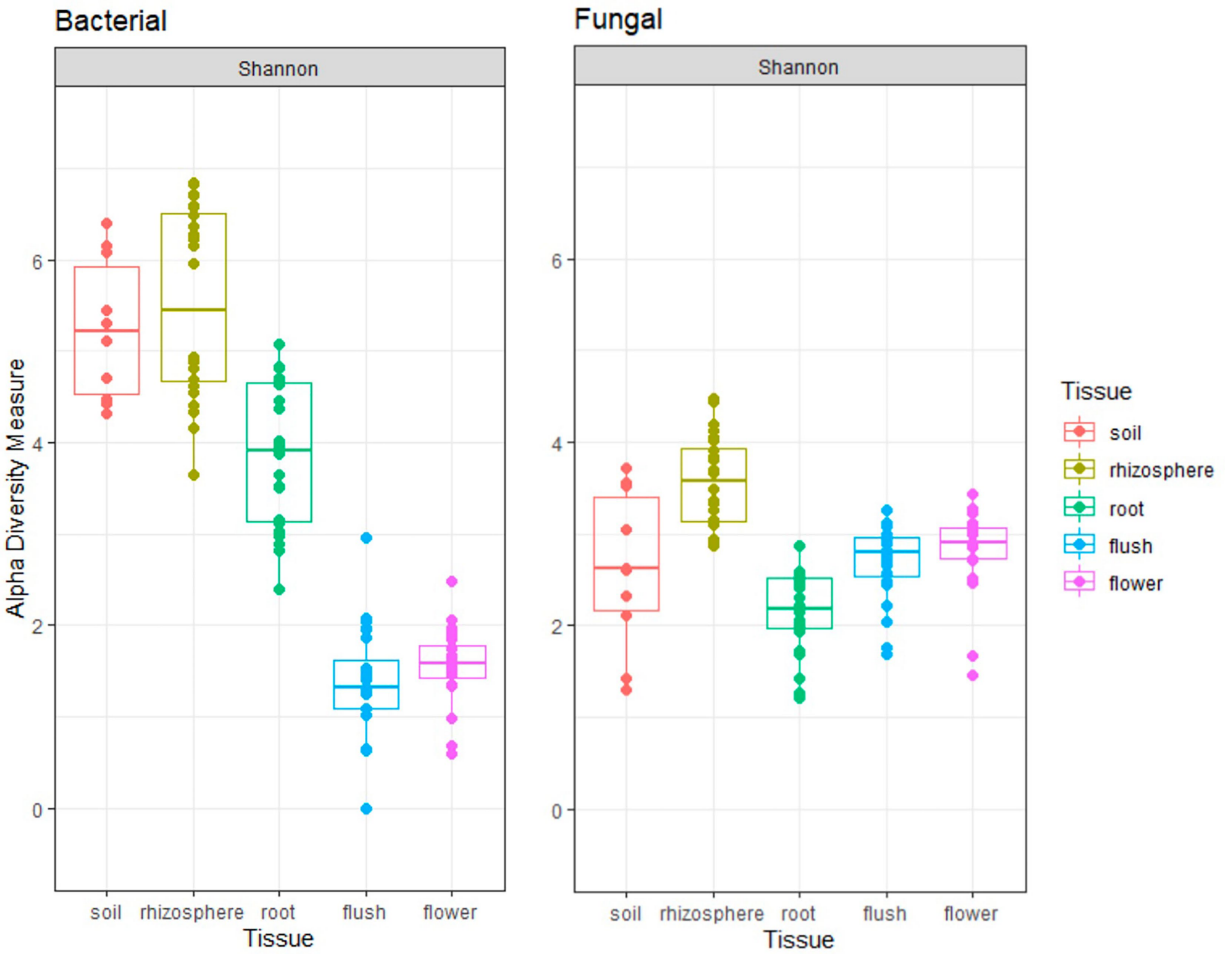
Shannon alpha-diversity plots indicate bacterial (left panel) and fungal (right panel) richness across five different citrus biocompartments (soil, rhizosphere, root, flush, and flower).

The Shannon index indicated that the rhizosphere had a significantly higher microbial richness among all plant tissue types for both bacteriome and mycobiome (*p* < 0.001 [pairwise Wilcox]; Figure 1). All the below ground bacteriome samples showed a significantly higher Shannon diversity index as compared to the above ground samples (*p* < 0.001 [pairwise Wilcox]). In contrast, the root mycobiome had a significantly lower fungal community richness of all tissue types (*p* < 0.001 [pairwise Wilcox]) and there was no significant difference with the soil fungal diversity (*p* = 0.25 [pairwise Wilcox]). The bacteriome richness was higher than the mycobiome richness in the below ground samples (soil, rhizosphere, and root), while the opposite was true for the above ground tissues (flush/flower). Flower and flush microbiome richness level were similar to each other in both groups. There was a year effect on the Shannon diversity index in the mycobiome communities (*p* < 0.05 [pairwise Wilcox]) but not in bacteriome (*p* = 0.23 [pairwise Wilcox]).

**Figure 2:**
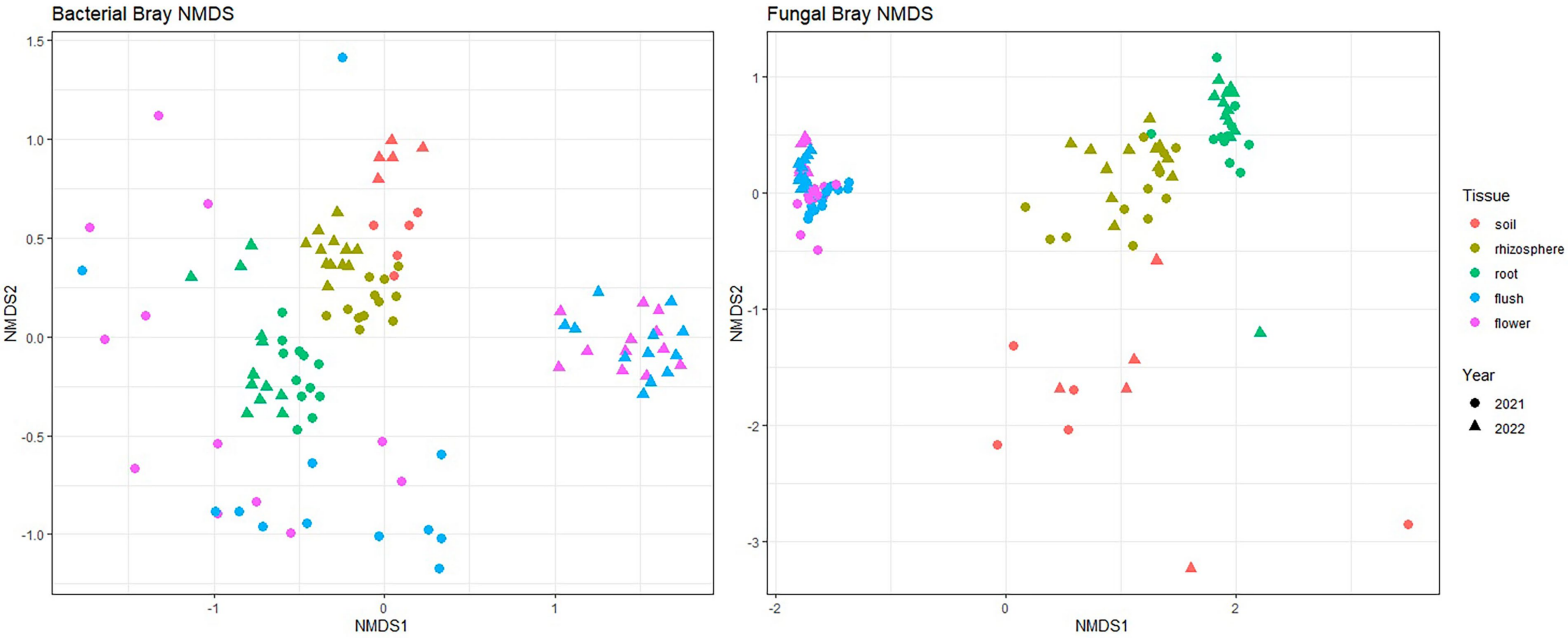
Bray–Curtis beta diversity for bacteria (left panel) and fungi (right panel) across five different citrus biocompartments (soil, rhizosphere, root, flush and flower). Points represent individual sample communities for one biocompartment from one citrus tree at 1 year. Points are colored by biocompartment and shaped by year collected.

Bray–Curtis beta-diversity metrics with NMDS were used to visualize how biocompartments impacted fungal and bacterial community composition (Figure 2). Our data indicated distinct clustering between above- and below-ground in both bacterial and fungal communities (*p* < 0.001 [Adonis]). Among belowground samples, clear clustering was measured for the soil, rhizosphere, and root in both bacterial and fungal groups. Among aboveground samples, flush and flower showed overlapping patterns in bacterial year-2 data and all fungal data. Year also had a significant effect (p < 0.001 [Adonis] for bacteriome; *p* < 0.05 [Adonis] for the mycobiome) in the clustering pattern, particularly for the bacterial flower and flush datasets.

**Figure 3:**
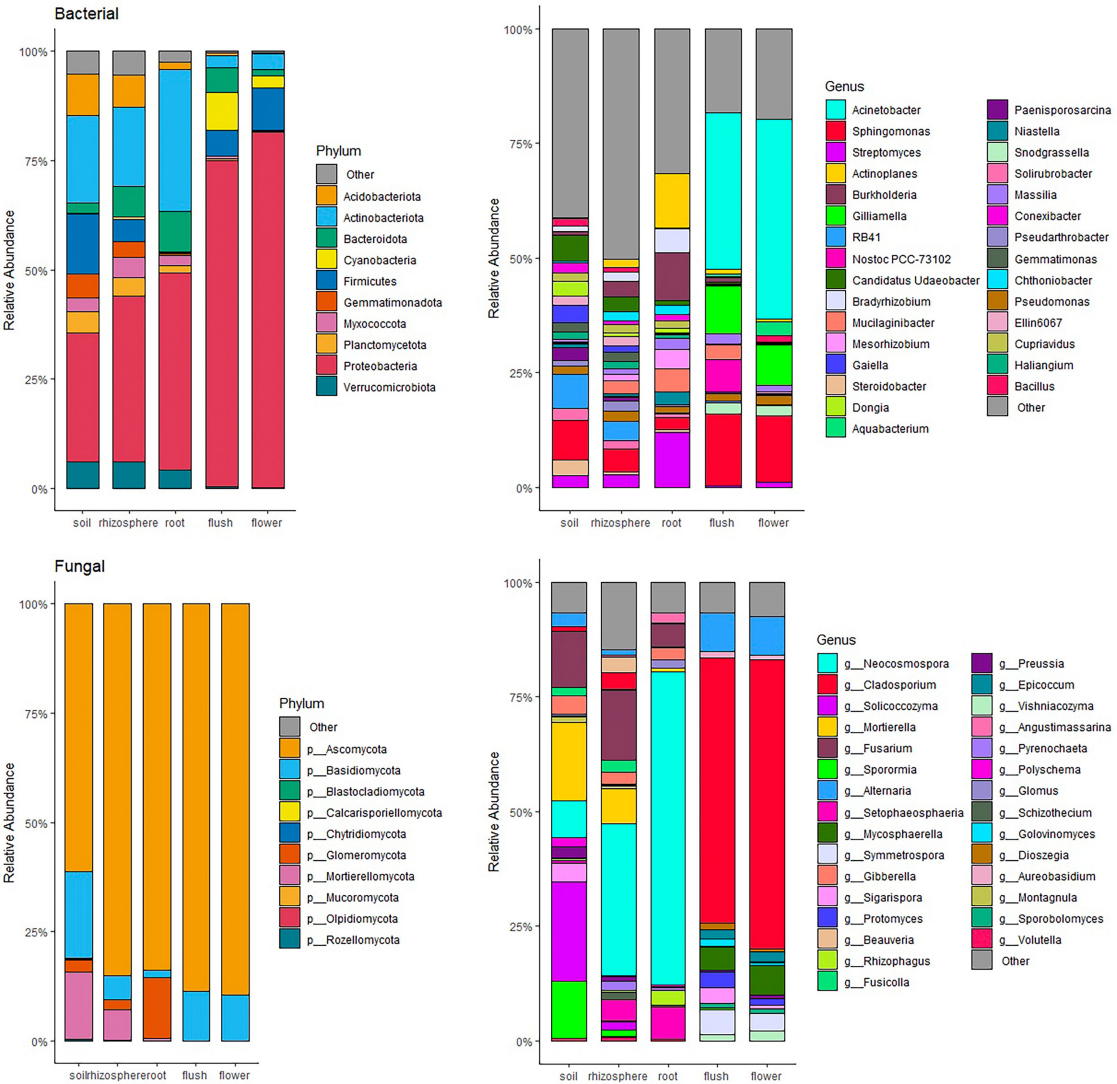
Relative abundant bar chat of bacteria (top panels) and fungi (bottom panels) community at phylum (left panels) and genus (right panels) level within individual citrus biocompartment (soil, rhizosphere, root, flush and flower). Only top 10 phyla and top 30 genera occurring at ≥ 1% relative abundance are displayed.

Proteobacteria and Ascomycota were the most abundant phyla within the entire dataset representing on average 47.7 and 81.6% of all taxa, respectively (Figure 3). Phyla Basidiomycota and Actinobacteria were also important phyla as they occurred in greater than 10% on average across the entire datasets. Several phyla with a relatively great abundance (greater than 5%) were unique to belowground or aboveground biocompartments. For example, Glomeromycota, Mortierellomycota, Acidobacteria, Gemmatimonadota, and Verrucomicrobiota were mainly found in soil, root and rhizosphere, whereas Cyanobacteria was only found in flower and flush samples. Although the most abundant phyla were the same in each tissue at the phylum level, differences were observed at the genus level especially between above and belowground. In the mycobiome, the most abundant genus in belowground samples was *Neocosmospora* (36.4%), while aboveground *Cladosporium* was the most dominant (60.4%). In the bacteriome, *Acinetobacter* was the most abundant genus in aboveground samples (38.7%), and the belowground samples were more diverse, with no single dominant genus.

**Figure 4:**
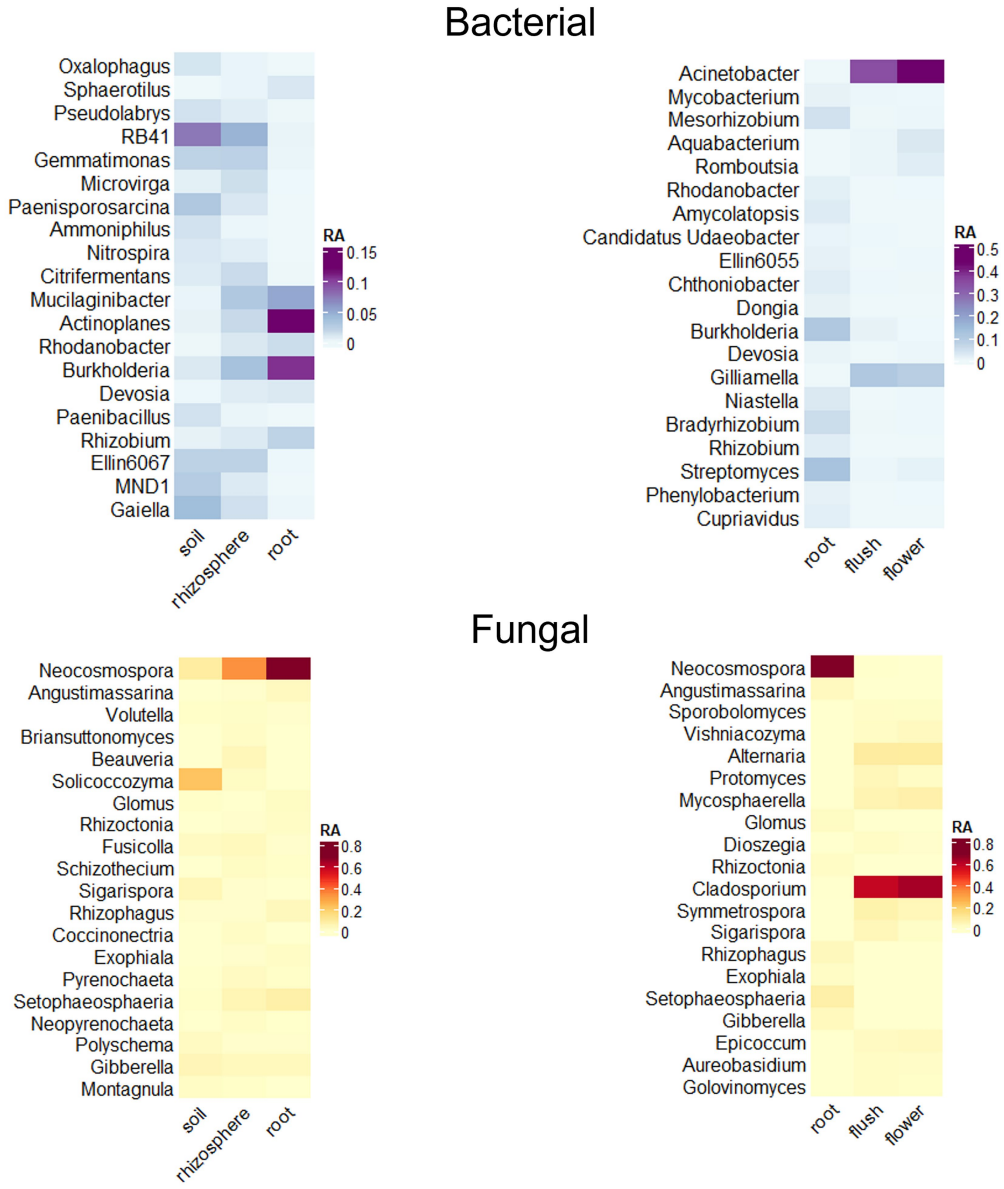
Relative abundance heat maps of significant taxa as determined through DESeq2 analyzes indicate recruitment of bacteria (top panels) and fungi (bottom panels) along the soil/rhizosphere/root endosphere axis (left panels) and signature taxa for the root/flush/flower (right panels) compartments. Data was filtered to a *p* < 0.01 cutoff and log2 fold change of > 5 or < −5. Only genera occurring at ≥ 1% relative abundance with the top 20 highest log2 fold changes are displayed.

We used DeSeq2 analyzes to indicate enrichment/rarefaction patterns of taxa along the soil, rhizosphere root axis and signature microbial taxa for the three plant biocompartments, root, flush and flower. We focused our analysis on the most prevalent and abundant taxa and applied a filtering metric that consisted of ≥ 50% incidence in roots and > 10% of bacterial flush and flower samples with a relative abundance > 1% (Figure 4). Our results indicated a root enrichment of several bacterial genera in the rhizosphere that were dominant in soil, but only a few of these were found in the root including *Actinoplanes*, *Burkholderia*, *Mucilaginibacter*, and *Rhizobium* and fungi *Glomus*, *Neocosmospora*, *Rhizophagus*, and *Setophaeosphaeria*. Several bacterial and fungal taxa were unique to roots and included the bacterial genera *Bradyrhizobium*, *Cupriavidus* and *Rhizobium* and the fungal genera *Glomus*, *Neocosmospora*, *Rhizophagus* and *Setophaeosphaeria*. In contrast, the bacterial genera *Acinetobacter*, *Aquabacterium*, *Gilliamella*, *Romboutsia* and fungal genera, *Alternaria*, *Aureobasidium*, *Cladosporium*, *Epicoccum*, *Mycospherella*, *Sigarispora*, and *Symmetrospora* were signature above ground taxa because only found in those compartments. Bacteria *Burkholderia* and *Streptomyces* were present in both below and aboveground tissue.

**Figure 5:**
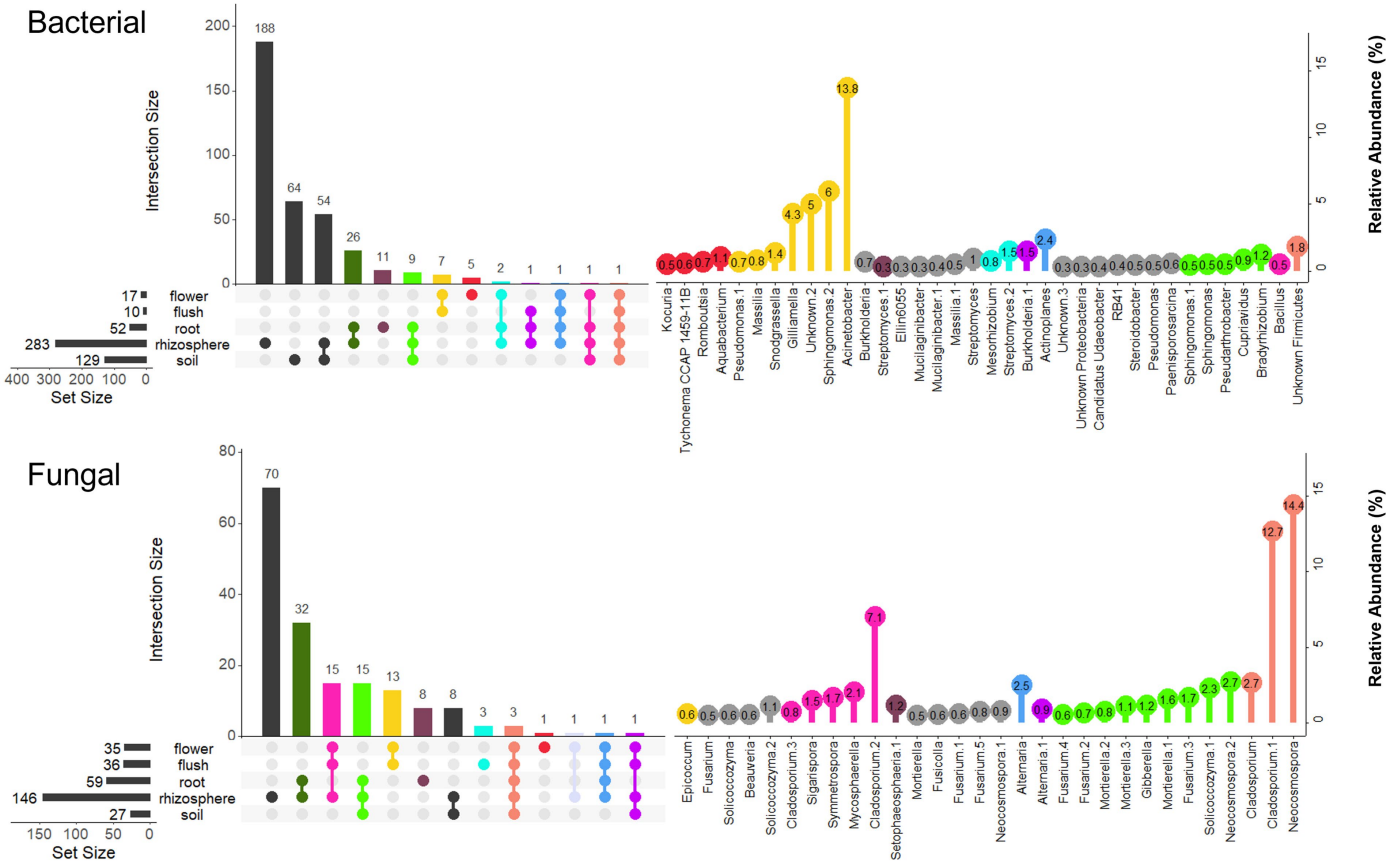
Prevalence Venn diagrams at the Amplicon Sequence Variant (ASV) level showing overlapping bacterial (top panel) and fungal (bottom panel) taxa that occur in ≥ 50% of the soil/rhizosphere/root samples and > 10% of the flush/flower samples within and across biocompartment. Only ASVs occurring at ≥ 0.5% relative abundance are displayed. Left panel represents the total number of ASVs for each biocompartment and intersection size for all biocomparment combinations. The right panel represents the ASV relative abundance (%) and color refers to the left panel and the biocompartment(s) where the ASVs were found.

The identity of the most prevalent ASVs that were unique to each biocompartment or shared across biocompartments were determined using Venn diagrams with a filtering consisting of ≥ 50% incidence for the belowground compartments and > 10% of bacterial flush and flower samples, with a relative abundance > 0.1% (Figure 5). This filtering narrowed the dataset to a total of 794 ASVs (491 bacterial and 303 fungal ASVs). The rhizosphere was the biocompartment with the highest number of unique filtered ASVs for both bacteria and fungi (429 ASVs total = 54%), whereas root, flower and flush only had 14, 6.5 and 5.8% of unique ASVs for the combined fungi and bacteria datasets, respectively. The fungal *Epicoccum* and bacterial *Acinetobacter*, *Aquabacterium*, *Gilliamella*, *Kocuria*, *Romboutsia*, *Snodgrassella*, *Tychonema* ASVs were biomarkers of the above ground tissues because they were only found in the flush, flower or both. The fungal *Beauveria*, *Fusarium*/*Giberella*/*Fusicola*, *Mortierella*, *Setophaeosphaeria*, *Solicoccozyma* and bacterial *Bradyrhizobium*, *Cupriavidus*, *Mucilagnibacter*, *Pseudathrobacter*, *Steroidobacter* ASVs were biomarkers of the belowground citrus because they were only found in the root, rhizosphere, or both. The majority (84%) of total number of fungal and bacterial ASVs inhabiting the roots (111 ASVs) were also found in the soil/rhizosphere suggesting they entered the root from the soil/rhizosphere. Only 3.4% of the total number bacterial and fungal ASVs (27 ASVs total) were capable of colonizing at least one of the below and above ground compartments highlighting their ubiquitous nature and included ASVs belonging to the fungal genera *Alternaria*, *Cladosporium*, *Mycosphaerella*, *Neocosmospora*, *Sigarispora*, and *Symmetrospora*, and the bacterial genera *Actinoplanes*, *Bacillus*, *Burkholderia*, *Firmicutes*, *Mesorhizobium*, *Pseudomonas*, *Massilia*, *Sphingomonas*, and *Streptomyces*.

## Discussion

The aim of this study was to characterize the citrus microbiome across five biocompartments (flush, flower, root, rhizosphere and bulk soil) during the floral development phase. At this stage, trees undergo drastic physiological shifts with respect to carbon reallocation, water dynamics and phytohormone production (Goldschmidt and Koch, 2017; Agustí et al., 2022) that are linked to significant shifts in microbial community assemblage (Ginnan et al., 2022). Both flower and flush are short-lived organs. Flowers host a unique set of microbes that may act as mediators of host reproduction and disease control (Burgess and Schaeffer, 2022). The flush is also a tender tissue fed on by several insect pests, including the Asian citrus psyllid (*Diaphorina citri*) vector of *C*Las, the causal bacterial agent of HLB (Hall and Albrigo, 2007), and profiling its microbiome could reveal potential biocontrol agents for HLB management. The citrus rhizosphere microbiome has also been a research focus because of its role in nutrient fixation, absorption and cycling as well as defense against pathogens (Xu et al., 2018; Ginnan et al., 2020; Zhang et al., 2021). Here we provide a better understanding of the acquisition of specific microbes along the soil-rhizosphere-root axis as plant endophytes may have bioactive functions relative to bioinoculant development (Lugtenberg et al., 2016; Santoyo et al., 2016).

The microbial biodiversity of citrus trees was primarily located in the plant rhizosphere, with the bacteriome showing higher taxonomic richness than the mycobiome (Blaustein et al., 2017; Ginnan et al., 2020). Trees were predominantly colonized across all compartments by Ascomycota fungi and Proteobacteria (Trivedi et al., 2010; Passera et al., 2018; Xu et al., 2018; Bai et al., 2019) but microbial composition within those groups was vastly different between the above and below ground compartments as indicated by beta diversity plots. Microbial diversity in the flush and flower was low and Ascomycota and Proteobacteria represented overwhelmingly 80% of the microbial relative abundance in those tissues. In contrast, belowground microbial assemblage was more complex especially for bacteria and included a wide range of taxonomic groups spanning across several bacterial phyla. Our data indicated that a minority of taxa (3.4%) were capable of colonizing both below and above ground habitats. We defined the core taxa of the citrus holobiont as genera prevalent in at least 50% of our samples and with a relative abundance of at least 1% and with ASVs within those groups capable of colonizing at least one below and above ground biocompartment. Based on these criteria we found that the fungi *Alternaria*, *Cladosporium*, *Fusarium* (syn. *Fusicolla, Gibberella*, *Neocosmospora*,), *Mycosphaerella*, *Sigarispora*, and *Symmetrospora*, and bacteria *Actinoplanes*, *Bacillus*, *Burkholderia*, *Firmicutes*, *Massilia*, *Mesorhizobium*, *Pseudomonas*, *Sphingomonas*, and *Streptomyces* represented core members of the citrus holobiont. Although additional sampling from orchards located in different citriculture areas may narrow that list. Many of these bacterial taxa are known plant growth promoters and biocontrol agents and can provide fitness advantage to the host (Lemanceau et al., 2017; Xu et al., 2018). However, the role of fungal taxa remains elusive. Several genera within the *Fusarium* species complex were found in our dataset (*Fusarium*, *Giberella*, *Fusicolla*, and *Neocosmospora*) and members within this group have a broad range of lifestyles including commensalism, mutualism, and parasitism (Crous et al., 2021). For example, *Fusarium solani* is a known pathogen of citrus causing wood dry rot (Sandoval-Denis et al., 2018) but the lifestyle of other species belonging to the *Fusarium* complex is unclear. Other fungal taxa within the citrus holobiont such as *Alternaria alternata*, *A. arborescens*, and *Mycosphaerella citri* were also reported to blemish fruits (Mondal et al., 2003; Wang et al., 2021). On the other hand, *Sigarispora* and *Symmetrospora* have been found in several habitats but with no known functions in citrus. Only *Cladosporium cladosporioides* was shown to inhibit *Liberibacter crescens*, a culturable surrogate of *C*Las (Blacutt et al., 2020), and could provide some benefits to the host. Deeper amplicon-based sequencing will help naming the fungal species associated with citrus which may provide some information about their lifestyle. Large scale sampling coupled with -omics technologies will shed light on the geographical distribution and functional attributes of the core fungal taxa within the citrus holobiont, although this approach remains limited by the availability of reference genomes (Xu et al., 2018).

The root-associated microbiome of healthy plants is a relatively stable ecosystem because roots are immersed in a buffered environment (the soil) that is not in under the direct constraints of extreme weather conditions and agricultural practices that above ground plant compartments experience. Roots are also less affected by the host phenological changes unlike flower and flush tissues (Ginnan et al., 2022). Root microbial assembly has been described as a two-step process, involving acquisition of specific microbes from the soil to the rhizosphere and a host-driven sorting step mechanism that subsets specific microbes into the root (Bulgarelli et al., 2013). Our DeSeq2 data clearly supported this mechanism in citrus, with enrichment of several organisms from the bulk soil to the rhizosphere, but with only few of these further capable of entering the citrus root endosphere (e.g., *Actinoplanes*, and *Burkholderia*). The microbiome of the rhizosphere was composed of the aforementioned core members of the citrus holobiont, plus signature underground taxa that included the bacterial genera *Bradirhizobium*, *Cupriavidus*, *Mucilaginibacter*, *Rhizobium* and *Steroidobater*, and fungal genera *Glomus*, and *Rhizophagus*. Comparative profiling of bulk soil and rhizosphere samples collected across distinct biogeographical regions from six continents also supported that these bacterial taxa were enriched in the rhizosphere (Xu et al., 2018). Root exudates act as signal molecules and food sources for the selective recruitment of microbes from bulk soil in exchange for increased nutrients assimilation and improved tolerance against abiotic and biotic stresses. Metagenomic sequencing of citrus soil and rhizosphere communities clearly showed that the functional traits enriched in the rhizosphere influenced microbial assembly and plant health (Xu et al., 2018). Specifically, enriched functional attributes affecting microbial assembly were involved in plant-microbe and microbe-microbe interactions (e.g., antimicrobial synthesis, biofilm formation), nutrient acquisition of microbes, and bioremediation of aromatic compounds. In addition, enriched functional traits that benefit the host were involved in nutrient acquisition, hormone balance, and pathogen inhibition (Xu et al., 2018).

Our data indicated that the microbial communities inhabiting the citrus root endosphere most likely originated from the rhizosphere (84% of ASVs) but with a threefold and fivefold decrease for both fungal and bacterial richness, respectively, which support previous findings (Reinhold-Hurek et al., 2015; Wang et al., 2020). The selective forces imposed by the plant host in the endorhiza are a bottleneck to biodiversity as observed in several plant systems (Fitzpatrick et al., 2018a; Deyett and Rolshausen, 2020; Zhang et al., 2021). Interestingly, the backbone of the root endospheric communities was comprised of taxa from the core rhizophere microbiome, suggesting that similar functional microbial traits overlap between the rhizosphere and root endosphere. We measured a strong enrichment pattern for some taxa including the bacteria *Actinoplanes*, *Burkholderia*, *Mucilaginibacter*, *Rhizobium*, *Rhodobacter*, and fungi *Glomus*, and *Rhizophagus*. All five bacteria can promote plant growth by either fixing nitrogen, solubilizing phosphorus, producing phytohormone production, and increasing abiotic stress tolerance as well as and protect against pathogens by producing antimicrobial compounds or priming plant defense (Santi et al., 2013; Zhang et al., 2017; Orsi et al., 2021; Boukhatem et al., 2022; Fan and Smith, 2022). *Glomus* and *Rhizophagus* are arbuscular mycorrhizal fungi (AMF) that commonly form symbiotic associations with the plant host, including citrus. AMF can facilitate water and nutrient acquisition (phosphorus and nitrogen) and support host defenses against pathogen attack (Hohmann and Messmer, 2017; Chen et al., 2018; Xi et al., 2022).

In contrast to rhizocompartments, above ground microorganisms associated with plants are under strong selective pressure because they are continually exposed to changing environmental conditions (rainfall, heat, and UV radiation) and agricultural practices (agrochemical sprays) but are also influenced by the host phenology (Vorholt, 2012; Burgess and Schaeffer, 2022; Ginnan et al., 2022). The strong year effect measured on bacteriome and mycobiome beta-diversity for above ground tissues clearly support the evidence that microbiome composition in flower and flush is volatile and under environmental constraints. The citrus flower and flush microbiome composition was very similar to the leaf and included both core taxa (*Acinetobacter*, *Romboutsia*, and *Sphingomonas*,) fulfilling community-stabilizing function and transient taxa (*Gilliamella* and *Snodgrassella*) with likely specialized function in the community (Ginnan et al., 2022). Interestingly, the fungus *Epicoccum* surfaced as a signature fungus capable of colonizing flush and flower. It was previously reported as inhibitory to *Liberibacter crescens* (Blacutt et al., 2020) and given those characteristics should be further explored as a potential biocontrol for HLB management. Other signature and ecologically important bacteria within the flush and flower microbiome included *Acinetobacter*, *Gilliamella*, *Snodgrassella*, and *Sphingomonas*. *Acinetobacter* is highly abundant in the floral nectar microbiome of *Citrus paradisi* and other plant species (Fridman et al., 2012; Alvarez-Perez and Herrera, 2013) and *Sphingomonas* has been reported as a frequent member of the citrus rhizosphere (Xu et al., 2018). Both bacteria have also been identified in the sap of other perennial hosts (Deyett and Rolshausen, 2020). *Snodgrassella* and *Gilliamella* are important members of the honeybee gut microbiome and have been speculated to be immigrant taxa introduced to the phyllosphere by pollinators during dispersal event (Powell et al., 2014; Ginnan et al., 2022). Bacteria can be introduced to plants by bees and potentially migrate from the flower to the vascular bundles resulting in systemic movement within the plant (Cellini et al., 2019; Kim et al., 2019). Together, this supports that members of the citrus microbiome can move acropetally and basipetally through the xylem and phloem (Compant et al., 2010; Deyett and Rolshausen, 2019, 2020). Abundance of these bacteria has been shown to peak at the flowering stage in citrus and grapevine (Deyett and Rolshausen, 2019; Ginnan et al., 2022). These bacteria have well known plant growth-promoting capabilities through phytohormone production, phosphate solubilization, and degradation of organometallic compounds and are also antagonistic toward pathogens (Liu et al., 2007; Kang et al., 2009, 2012; Asaf et al., 2020). It is tempting to speculate that similar the rhizosphere, microbial recruitment mechanisms of beneficial bacteria also occur in the phyllosphere to provide the host with exogenous services and promote reproductive and vegetative cycles in sync with the host phenology.

## Conclusion

This study provides new information about assemblage of microbial communities in citrus. Our results from a single orchard support that the citrus microbiome is composed of core taxonomic groups that are mainly of soil origin and that can systemically colonize trees. There is also evidence of a microbial niche compartmentalization with specialized taxa capable of colonizing either the above or the below ground biocompartments. Our findings support that transient taxa, whose colonization patterns are in sync with the host phenology, are abundant during flowering and tree flushing. We identified putative plant growth promoting bacteria (e.g., *Burkholderia*, *Sphingomonas*, and *Streptomyces*) enriched in all biocomparments that could be harnessed for bioproduct commercialization to improve tree health. We also identify tissue specific microbes (e.g., *Acinetobacter* and *Epicoccum*) that could colonize the citrus flush and flower and could enhance tree productivity or management against pests and diseases and notably HLB. Broad biogeographical sampling and shotgun metagenomic approach have greatly helped comprehend the structural and functional composition of the citrus rhizosphere microbiome. The next frontier is to expand this approach to the plant endosphere because it could harbor host-selected microbes with bioactive functions. Understanding in what capacity beneficial microbes respond to citricultural practices will help developing recommendations to improve fertilization and pest and disease management programs. These research efforts will narrow the search for active biofertilizers and biopesticides that could be commercialized by agrochemical companies into new green technologies.

## Data availability statement

The datasets presented in this study can be found in online repositories. The names of the repository/repositories and accession number(s) can be found at: https://www.ncbi.nlm.nih.gov/, SUB12502574 and SUB12495203.

## Author contributions

MX: methodology, data collection and analysis, writing original manuscript draft, review and editing. ED: data analysis, scientific guidance and manuscript review. JS: scientific guidance and manuscript review. AK: project logistics and data collection. MR: secure funding, manuscript review and editing. PR: project conceptualization, secure funding, methodology, data collection and analysis, writing original manuscript draft, and review and editing. All authors contributed to the article and approved the submitted version.

## Funding

This work was supported by USDA NIFA grant no. 2017-70016-26053 and 2020-70029-33202, CDFA grant no. SCB16056 and 19-0001-034-SF, USDA National Institute of Food and Agriculture Hatch Projects CA-R-PPA-5020-H and CA-R-BPS-5071-H.

## Conflict of interest

The authors declare that the research was conducted in the absence of any commercial or financial relationships that could be construed as a potential conflict of interest.

## Publisher’s note

All claims expressed in this article are solely those of the authors and do not necessarily represent those of their affiliated organizations, or those of the publisher, the editors and the reviewers. Any product that may be evaluated in this article, or claim that may be made by its manufacturer, is not guaranteed or endorsed by the publisher.

## Footnotes

^1^ http://www.earthmicrobiome.org/

## References

[ref1] Agustí M. Mesejo C. Muñoz-Fambuena N. Vera-Sirera F. de Lucas M. Martínez-Fuentes A.. (2020). Fruit-dependent epigenetic regulation of flowering in citrus. New Phytol. 225, 376–384. doi: 10.1111/nph.16044, PMID: 31273802

[ref2] Agustí M. Reig C. Martínez-Fuentes A. Mesejo C. (2022). Advances in citrus flowering: a review. Front. Plant Sci. 13:868831. doi: 10.3389/fpls.2022.868831, PMID: 35463419 PMC9024417

[ref3] Aleklett K. Hart M. Shade A. (2014). The microbial ecology of flowers: an emerging frontier in phyllosphere research. Botany 92, 253–266. doi: 10.1139/cjb-2013-0166

[ref4] Alvarez-Perez S. Herrera C. M. (2013). Composition, richness and nonrandom assembly of culturable bacterial–microfungal communities in floral nectar of Mediterranean plants. FEMS Microbiol. Ecol. 83, 685–699. doi: 10.1111/1574-6941.12027, PMID: 23057414

[ref5] Asaf S. Numan M. Khan A. L. Al-Harrasi A. (2020). Sphingomonas: from diversity and genomics to functional role in environmental remediation and plant growth. Crit. Rev. Biotechnol. 40, 138–152. doi: 10.1080/07388551.2019.1709793, PMID: 31906737

[ref6] Bai Y. Wang J. Jin L. Zhan Z. Guan L. Zheng G.. (2019). Deciphering bacterial community variation during soil and leaf treatments with biologicals and biofertilizers to control huanglongbing in citrus trees. J. Phytopathol. 167, 686–694. doi: 10.1111/jph.12860

[ref7] Blacutt A. Ginnan N. Dang T. Bodaghi S. Vidalakis G. Ruegger P.. (2020). An in vitro pipeline for screening and selection of citrus-associated microbiota with potential anti-"Candidatus liberibacter asiaticus" properties. Appl. Environ. Microbiol. 86, 1–18. doi: 10.1128/AEM.02883-19, PMID: 32086307 PMC7117939

[ref8] Blaustein R. A. Lorca G. L. Meyer J. L. Gonzalez C. F. Teplitski M. (2017). Defining the core citrus leaf-and root-associated microbiota: factors associated with community structure and implications for managing huanglongbing (citrus greening) disease. Appl. Environ. Microbiol. 83:e00210-17. doi: 10.1128/AEM.00210-17, PMID: 28341678 PMC5440699

[ref9] Bodenhausen N. Horton M. W. Bergelson J. (2013). Bacterial communities associated with the leaves and the roots of Arabidopsis thaliana. PLoS One 8:e56329. doi: 10.1371/journal.pone.0056329, PMID: 23457551 PMC3574144

[ref10] Boukhatem Z. F. Merabet C. Tsaki H. (2022). Plant growth promoting actinobacteria, the most promising candidates as bioinoculants? Front. Agron. 4:14. doi: 10.3389/fagro.2022.849911

[ref11] Bové J. M. (2006). Huanglongbing: a destructive, newly-emerging, century-old disease of citrus. J. Plant Pathol. 88, 7–37.

[ref12] Bulgarelli D. Rott M. Schlaeppi K. Loren V. van Themaat E. Ahmadinejad N.. (2012). Revealing structure and assembly cues for Arabidopsis root-inhabiting bacterial microbiota. Nature 488, 91–95. doi: 10.1038/nature11336, PMID: 22859207

[ref13] Bulgarelli D. Schlaeppi K. Spaepen S. Van Themaat E. V. L. Schulze-Lefert P. (2013). Structure and functions of the bacterial microbiota of plants. Annu. Rev. Plant Biol. 64, 807–838. doi: 10.1146/annurev-arplant-050312-120106 23373698

[ref14] Burgess E. C. Schaeffer R. N. (2022). The floral microbiome and its management in agroecosystems: a perspective. J. Agric. Food Chem. 70, 9819–9825. doi: 10.1021/acs.jafc.2c02037, PMID: 35917340

[ref15] Callahan B. J. McMurdie P. J. Rosen M. J. Han A. W. Johnson A. J. A. Holmes S. P. (2016). DADA2: high-resolution sample inference from Illumina amplicon data. Nat. Methods 13, 581–583. doi: 10.1038/nmeth.3869, PMID: 27214047 PMC4927377

[ref16] Caporaso J. G. Kuczynski J. Stombaugh J. Bittinger K. Bushman F. D. Costello E. K.. (2010). QIIME allows analysis of high-throughput community sequencing data. Nat. Methods 7, 335–336. doi: 10.1038/nmeth.f.303, PMID: 20383131 PMC3156573

[ref17] CDFA (2021). California Agricultural Production Statistics. California: CDFA.

[ref18] Cellini A. Giacomuzzi V. Donati I. Farneti B. Rodriguez-Estrada M. T. Savioli S.. (2019). Pathogen-induced changes in floral scent may increase honeybee-mediated dispersal of Erwinia amylovora. ISME J. 13, 847–859. doi: 10.1038/s41396-018-0319-2, PMID: 30504898 PMC6461938

[ref19] Chen M. Arato M. Borghi L. Nouri E. Reinhardt D. (2018). Beneficial services of arbuscular mycorrhizal fungi–from ecology to application. Front. Plant Sci. 9:1270. doi: 10.3389/fpls.2018.01270, PMID: 30233616 PMC6132195

[ref20] Chiyaka C. Singer B. H. Halbert S. E. Morris J. G. Jr. van Bruggen A. H. C. (2012). Modeling huanglongbing transmission within a citrus tree. Proc. Natl. Acad. Sci. 109, 12213–12218. doi: 10.1073/pnas.1208326109, PMID: 22783015 PMC3409777

[ref21] Compant S. Clément C. Sessitsch A. (2010). Plant growth-promoting bacteria in the rhizo-and endosphere of plants: their role, colonization, mechanisms involved and prospects for utilization. Soil Biol. Biochem. 42, 669–678. doi: 10.1016/j.soilbio.2009.11.024

[ref22] Crous P. W. Lombard L. Sandoval-Denis M. Seifert K. A. Schroers H.-J. Chaverri P.. (2021). *Fusarium*: more than a node or a foot-shaped basal cell. Stud. Mycol. 98:100116. doi: 10.1016/j.simyco.2021.100116, PMID: 34466168 PMC8379525

[ref23] Cui Z. Huntley R. B. Zeng Q. Steven B. (2021). Temporal and spatial dynamics in the apple flower microbiome in the presence of the phytopathogen *Erwinia amylovora*. ISME J. 15, 318–329. doi: 10.1038/s41396-020-00784-y, PMID: 33024293 PMC7853089

[ref24] Deyett E. Rolshausen P. E. (2019). Temporal dynamics of the sap microbiome of grapevine under high Pierce’s disease pressure. Front. Plant Sci. 10:1246. doi: 10.3389/fpls.2019.01246, PMID: 31681363 PMC6805966

[ref25] Deyett E. Rolshausen P. E. (2020). Endophytic microbial assemblage in grapevine. FEMS Microbiol. Ecol. 96:fiaa053. doi: 10.1093/femsec/fiaa053, PMID: 32196076

[ref26] Etxeberria E. Gonzalez P. Achor D. Albrigo G. (2009). Anatomical distribution of abnormally high levels of starch in HLB-affected Valencia orange trees. Physiol. Mol. Plant Pathol. 74, 76–83. doi: 10.1016/j.pmpp.2009.09.004

[ref27] Fan D. Smith D. L. (2022). Mucilaginibacter sp. K improves growth and induces salt tolerance in nonhost plants via multilevel mechanisms. Front. Plant Sci. 13. doi: 10.3389/fpls.2022.938697, PMID: 35832221 PMC9271937

[ref28] Fitzpatrick C. R. Copeland J. Wang P. W. Guttman D. S. Kotanen P. M. Johnson M. T. J. (2018a). Assembly and ecological function of the root microbiome across angiosperm plant species. Proc. Natl. Acad. Sci. 115, E1157–E1165. doi: 10.1073/pnas.1717617115 29358405 PMC5819437

[ref29] Fitzpatrick C. R. Lu-Irving P. Copeland J. Guttman D. S. Wang P. W. Baltrus D. A.. (2018b). Chloroplast sequence variation and the efficacy of peptide nucleic acids for blocking host amplification in plant microbiome studies. Microbiome 6, 1–10. doi: 10.1186/s40168-018-0534-0 30121081 PMC6098832

[ref30] Fridman S. Izhaki I. Gerchman Y. Halpern M. (2012). Bacterial communities in floral nectar. Environ. Microbiol. Rep. 4, 97–104. doi: 10.1111/j.1758-2229.2011.00309.x 23757235

[ref31] Ginnan N. A. Dang T. Bodaghi S. Ruegger P. M. McCollum G. England G.. (2020). Disease-induced microbial shifts in citrus indicate microbiome-derived responses to huanglongbing across the disease severity spectrum. Phytobiomes J. 4, 375–387. doi: 10.1094/PBIOMES-04-20-0027-R

[ref32] Ginnan N. A. de Anda N. I. Campos Freitas Vieira F. Rolshausen P. E. Roper M. C. (2022). Microbial turnover and dispersal events occur in synchrony with plant phenology in the perennial Evergreen tree crop Citrus sinensis. MBio 13, e00343–e00322. doi: 10.1128/mbio.00343-22, PMID: 35642946 PMC9239260

[ref33] Goldschmidt E. E. Koch K. E. (2017). “Citrus,” in Photoassimilate Distribution Plants and Crops Source-Sink Relationships. eds. Zamsky E. Schaffer A. E. (New York: Routledge), 797–824.

[ref34] Gopal M. Gupta A. Thomas G. V. (2013). Bespoke microbiome therapy to manage plant diseases. Front. Microbiol. 4:355. doi: 10.3389/fmicb.2013.00355 24348466 PMC3847548

[ref35] Gottel N. R. Castro H. F. Kerley M. Yang Z. Pelletier D. A. Podar M.. (2011). Distinct microbial communities within the endosphere and rhizosphere of Populus deltoides roots across contrasting soil types. Appl. Environ. Microbiol. 77, 5934–5944. doi: 10.1128/AEM.05255-11, PMID: 21764952 PMC3165402

[ref36] Hall D. G. Albrigo L. G. (2007). Estimating the relative abundance of flush shoots in citrus with implications on monitoring insects associated with flush. HortScience 42, 364–368. doi: 10.21273/HORTSCI.42.2.364

[ref37] Hohmann P. Messmer M. M. (2017). Breeding for mycorrhizal symbiosis: focus on disease resistance. Euphytica 213:113. doi: 10.1007/s10681-017-1900-x

[ref38] Kang S.-M. Joo G.-J. Hamayun M. Na C.-I. Shin D.-H. Kim H. Y.. (2009). Gibberellin production and phosphate solubilization by newly isolated strain of *Acinetobacter calcoaceticus* and its effect on plant growth. Biotechnol. Lett. 31, 277–281. doi: 10.1007/s10529-008-9867-2, PMID: 18931973

[ref39] Kang S.-M. Khan A. L. Hamayun M. Shinwari Z. K. Kim Y.-H. Joo G.-J.. (2012). Acinetobacter calcoaceticus ameliorated plant growth and influenced gibberellins and functional biochemicals. Pak. J. Bot. 44, 365–372.

[ref40] Kim D.-R. Cho G. Jeon C.-W. Weller D. M. Thomashow L. S. Paulitz T. C.. (2019). A mutualistic interaction between Streptomyces bacteria, strawberry plants and pollinating bees. Nat. Commun. 10, 1–10. doi: 10.1038/s41467-019-12785-3 31641114 PMC6805876

[ref41] Lau J. A. Lennon J. T. (2011). Evolutionary ecology of plant–microbe interactions: soil microbial structure alters selection on plant traits. New Phytol. 192, 215–224. doi: 10.1111/j.1469-8137.2011.03790.x, PMID: 21658184

[ref42] Lemanceau P. Blouin M. Muller D. Moënne-Loccoz Y. (2017). Let the core microbiota be functional. Trends Plant Sci. 22, 583–595. doi: 10.1016/j.tplants.2017.04.008, PMID: 28549621

[ref43] Li S. Wu F. Duan Y. Singerman A. Guan Z. (2020). Citrus greening: management strategies and their economic impact. HortScience 55, 604–612. doi: 10.21273/HORTSCI14696-19

[ref44] Liu C. H. Chen X. Liu T. T. Lian B. Gu Y. Caer V.. (2007). Study of the antifungal activity of Acinetobacter baumannii LCH001 in vitro and identification of its antifungal components. Appl. Microbiol. Biotechnol. 76, 459–466. doi: 10.1007/s00253-007-1010-0, PMID: 17534613

[ref45] Lu T. Ke M. Lavoie M. Jin Y. Fan X. Zhang Z.. (2018). Rhizosphere microorganisms can influence the timing of plant flowering. Microbiome 6, 1–12. doi: 10.1186/s40168-018-0615-0 30587246 PMC6307273

[ref46] Lugtenberg B. J. J. Caradus J. R. Johnson L. J. (2016). Fungal endophytes for sustainable crop production. FEMS Microbiol. Ecol 92:92. doi: 10.1093/femsec/fiw194 27624083

[ref47] Lundberg D. S. Lebeis S. L. Paredes S. H. Yourstone S. Gehring J. Malfatti S.. (2012). Defining the core *Arabidopsis thaliana* root microbiome. Nature 488, 86–90. doi: 10.1038/nature11237, PMID: 22859206 PMC4074413

[ref48] McMurdie P. J. Holmes S. (2013). Phyloseq: an R package for reproducible interactive analysis and graphics of microbiome census data. PLoS One 8:e61217. doi: 10.1371/journal.pone.0061217, PMID: 23630581 PMC3632530

[ref49] Mondal S. N. Gottwald T. R. Timmer L. W. (2003). Environmental factors affecting the release and dispersal of ascospores of *Mycosphaerella citri*. Phytopathology 93, 1031–1036. doi: 10.1094/PHYTO.2003.93.8.1031, PMID: 18943870

[ref50] Moss G. I. (1969). Influence of temperature and photoperiod on flower induction and inflorescence development in sweet orange (*Citrus sinensis* L. Osbeck). J. Hortic. Sci. 44, 311–320. doi: 10.1080/00221589.1969.11514314

[ref51] Moss G. I. (1976). Temperature effects on flower initiation in sweet orange (*Citrus sinensis*). Aust. J. Agric. Res. 27, 399–407. doi: 10.1071/AR9760399

[ref52] National Research Council (2010). Strategic Planning for the Florida Citrus Industry: Addressing Citrus Greening Disease. Washington, DC: National Academies Press.

[ref53] Olesen T. Smith G. Muldoon S. J. (2013). Flush development in Tahitian lime. Aust. J. Bot. 61, 358–364. doi: 10.1071/BT13104

[ref54] Orsi E. Beekwilder J. Eggink G. Kengen S. W. M. Weusthuis R. A. (2021). The transition of Rhodobacter sphaeroides into a microbial cell factory. Biotechnol. Bioeng. 118, 531–541. doi: 10.1002/bit.27593, PMID: 33038009 PMC7894463

[ref55] Passera A. Alizadeh H. Azadvar M. Quaglino F. Alizadeh A. Casati P.. (2018). Studies of microbiota dynamics reveals association of “Candidatus Liberibacter asiaticus” infection with citrus (*Citrus sinensis*) decline in south of Iran. Int. J. Mol. Sci. 19:1817. doi: 10.3390/ijms19061817, PMID: 29925799 PMC6032414

[ref56] Peiffer J. A. Spor A. Koren O. Jin Z. Tringe S. G. Dangl J. L.. (2013). Diversity and heritability of the maize rhizosphere microbiome under field conditions. Proc. Natl. Acad. Sci. 110, 6548–6553. doi: 10.1073/pnas.1302837110, PMID: 23576752 PMC3631645

[ref57] Powell J. E. Martinson V. G. Urban-Mead K. Moran N. A. (2014). Routes of acquisition of the gut microbiota of the honeybee *Apis mellifera*. Appl. Environ. Microbiol. 80, 7378–7387. doi: 10.1128/AEM.01861-14, PMID: 25239900 PMC4249178

[ref58] Rastogi G. Sbodio A. Tech J. J. Suslow T. V. Coaker G. L. Leveau J. H. J. (2012). Leaf microbiota in an agroecosystem: spatiotemporal variation in bacterial community composition on field-grown lettuce. ISME J. 6, 1812–1822. doi: 10.1038/ismej.2012.32, PMID: 22534606 PMC3446804

[ref59] Redford A. J. Fierer N. (2009). Bacterial succession on the leaf surface: a novel system for studying successional dynamics. Microb. Ecol. 58, 189–198. doi: 10.1007/s00248-009-9495-y, PMID: 19221834

[ref60] Reinhold-Hurek B. Bünger W. Burbano C. S. Sabale M. Hurek T. (2015). Roots shaping their microbiome: global hotspots for microbial activity. Annu. Rev. Phytopathol. 53, 403–424. doi: 10.1146/annurev-phyto-082712-102342, PMID: 26243728

[ref61] Sagaram U. S. DeAngelis K. M. Trivedi P. Andersen G. L. Lu S.-E. Wang N. (2009). Bacterial diversity analysis of Huanglongbing pathogen-infected citrus, using PhyloChip arrays and 16S rRNA gene clone library sequencing. Appl. Environ. Microbiol. 75, 1566–1574. doi: 10.1128/AEM.02404-08, PMID: 19151177 PMC2655442

[ref62] Sandoval-Denis M. Guarnaccia V. Polizzi G. Crous P. W. (2018). Symptomatic citrus trees reveal a new pathogenic lineage in Fusarium and two new Neocosmospora species. Persoonia-Mol. Phylogeny Evol. Fungi 40, 1–25. doi: 10.3767/persoonia.2018.40.01, PMID: 30504994 PMC6146640

[ref63] Santi C. Bogusz D. Franche C. (2013). Biological nitrogen fixation in non-legume plants. Ann. Bot. 111, 743–767. doi: 10.1093/aob/mct048, PMID: 23478942 PMC3631332

[ref64] Santoyo G. Moreno-Hagelsieb G. del Carmen Orozco-Mosqueda M. Glick B. R. (2016). Plant growth-promoting bacterial endophytes. Microbiol. Res. 183, 92–99. doi: 10.1016/j.micres.2015.11.008 26805622

[ref65] Schlaeppi K. Bulgarelli D. (2015). The plant microbiome at work. Mol. Plant-Microbe Interact. 28, 212–217. doi: 10.1094/MPMI-10-14-0334-FI 25514681

[ref66] Shade A. Handelsman J. (2012). Beyond the Venn diagram: the hunt for a core microbiome. Environ. Microbiol. 14, 4–12. doi: 10.1111/j.1462-2920.2011.02585.x, PMID: 22004523

[ref67] Stander J. O. P. J. (2015). The reproductive phenology of citrus III: Morphogenesis from flower to fruit. Technology, 77–83.

[ref68] Trivedi P. Duan Y. Wang N. (2010). Huanglongbing, a systemic disease, restructures the bacterial community associated with citrus roots. Appl. Environ. Microbiol. 76, 3427–3436. doi: 10.1128/AEM.02901-09, PMID: 20382817 PMC2876436

[ref69] Vorholt J. A. (2012). Microbial life in the phyllosphere. Nat. Rev. Microbiol. 10, 828–840. doi: 10.1038/nrmicro2910 23154261

[ref70] Wang F. Saito S. Michailides T. J. Xiao C.-L. (2021). Phylogenetic, morphological, and pathogenic characterization of Alternaria species associated with fruit rot of mandarin in California. Plant Dis. 105, 2606–2617. doi: 10.1094/PDIS-10-20-2145-RE, PMID: 33373282

[ref71] Wang X. Wang M. Xie X. Guo S. Zhou Y. Zhang X.. (2020). An amplification-selection model for quantified rhizosphere microbiota assembly. Sci. Bull. 65, 1436–1439. doi: 10.1016/j.scib.2020.04.041 36659026

[ref72] Weinert N. Piceno Y. Ding G.-C. Meincke R. Heuer H. Berg G.. (2011). PhyloChip hybridization uncovered an enormous bacterial diversity in the rhizosphere of different potato cultivars: many common and few cultivar-dependent taxa. FEMS Microbiol. Ecol. 75, 497–506. doi: 10.1111/j.1574-6941.2010.01025.x, PMID: 21204872

[ref01] Wickham H. (2016). ggplot2-Elegant Graphics for Data Analysis. Cham, Switz: Springer International Publishing.

[ref73] Wu Y. Qu M. Pu X. Lin J. Shu B. (2020). Distinct microbial communities among different tissues of citrus tree *Citrus reticulata* cv. Chachiensis. Sci. Rep 10:6068. doi: 10.1038/s41598-020-62991-z, PMID: 32269258 PMC7142118

[ref74] Xi M. Deyett E. Ginnan N. Ashworth V. E. T. M. Dang T. Bodaghi S.. (2022). Arbuscular mycorrhizal fungal composition across US citrus orchards, management strategies, and disease severity spectrum. *BioRxiv* [Epub ahead of preprint]. doi: 10.1101/2022.03.01.482593

[ref75] Xu J. Zhang Y. Zhang P. Trivedi P. Riera N. Wang Y.. (2018). The structure and function of the global citrus rhizosphere microbiome. Nat. Commun. 9:4894. doi: 10.1038/s41467-018-07343-2, PMID: 30459421 PMC6244077

[ref76] Zhang Y. Trivedi P. Xu J. Caroline Roper M. Wang N. (2021). The citrus microbiome: from structure and function to microbiome engineering and beyond. Phytobiomes J. 5, 249–262. doi: 10.1094/PBIOMES-11-20-0084-RVW

[ref77] Zhang Y. Xu J. Riera N. Jin T. Li J. Wang N. (2017). Huanglongbing impairs the rhizosphere-to-rhizoplane enrichment process of the citrus root-associated microbiome. Microbiome 5:97. doi: 10.1186/s40168-017-0304-4, PMID: 28797279 PMC5553657

